# The Resistance Mechanisms of Anilinopyrimidine Fungicide Pyrimethanil in *Sclerotinia sclerotiorum*

**DOI:** 10.3390/jof11050344

**Published:** 2025-04-28

**Authors:** Yanfen Wang, Zeyuan Chen, Tiancheng Liu, Xupeng Gao, Yanchao Shi, Honghui Wu, Runqiang Liu, Yunchao Kan, Hao Yu, Feng Zhou

**Affiliations:** School of Plant Protection and Environment, Henan Institute of Science and Technology, Xinxiang 453003, China; wyfhist@163.com (Y.W.); czy0544108@163.com (Z.C.); ltc8610@163.com (T.L.); 17630890519@163.com (X.G.); syc16788@163.com (Y.S.); 17344663967@163.com (H.W.); liurunqiang1983@126.com (R.L.); kanyunchao@163.com (Y.K.)

**Keywords:** *Sclerotinia sclerotiorum*, pyrimethanil, resistance mechanism, cystathione-γ synthase

## Abstract

The necrotrophic pathogen *Sclerotinia sclerotiorum* is widely distributed and infects a broad range of hosts, making it one of the most economically damaging plant pathogens. This study demonstrated that pyrimethanil, an anilinopyrimidine fungicide, exhibited potent activity against *S. sclerotiorum*, with EC_50_ values ranging from 0.411 to 0.610 μg/mL. Four highly pyrimethanil-resistant mutants were obtained through chemical taming, with EC_50_ values of 7.247 to 24.718 μg/mL. These mutants exhibited significantly reduced mycelial growth, sclerotia production, and pathogenicity compared to their wild-type parental isolates, indicating that pyrimethanil resistance suffered a fitness penalty in *S. sclerotiorum*. Notably, three mutants (DDJH-Pyri-R1, DDJH-Pyri-R3, and DDJH-Pyri-R4), completely lose the capacity to infect detached tomato leaves. Point mutations that cause amino acid changes in the predicted sequence of cystathione-γ synthase (CGS) and cystathione-β lyase (CBL), encoded by *SsCGS1* and *SsCGS2*, were identified in three mutants. However, one mutant (DDJH-Pyri-R2) showed no mutations in these genes, suggesting an alternative resistance mechanism. Molecular docking revealed that mutations in SsCGS1-R3, SsCGS1-R4, and SsCGS2-R1 reduced the binding affinity between pyrimethanil and SsCGSs. No cross-resistance was observed between pyrimethanil and other commonly used fungicides, including carbendazim, fludioxonil, prochloraz, tebuconazole, pyraclostrobin, boscalid, fluazinam, and cyprodinil. These findings provide valuable insights for designing resistance inhibitors and suggest that pyrimethanil has significant potential for controlling soybean sclerotinia stem rot (SSR) caused by *S. sclerotiorum*.

## 1. Introduction

Soybean (*Glycine max*) is an extremely important oilseed crop that provides a source of both vegetable protein and cooking oil for people throughout the world [1,2]. In China, which is the world’s largest soybean consumer and importer, soybean is regarded as a critical resource for maintaining food security [3]. According to recent statistics, domestic soybean production in China has grown to approximately 15 million tons per year during the previous decade [4]. However, soybean sclerotinia stem rot (SSR), which is caused by the fungal pathogen *Sclerotinia sclerotiorum*, can cause significant damage to soybean plants, and is a constant threat to crop production as it attacks both seedlings and adult plants, as well as the flowering stages of growth [4]. The damage caused by this devastating disease generally results in a 20–30% reduction in yield, but outbreaks can result in losses of more than 50%, or even complete crop failure [5]. In the absence of resistant varieties, the prevention and control of SSR depends primarily on the application of chemical fungicides [6].

Pyrimethanil, a broad-spectrum anilinopyrimidine fungicide, was first registered in the USA in 2004 for post-harvest use on pome fruits. It is also effective as a seed treatment and foliar spray, offering both protective and curative actions [7]. Anilinopyrimidine fungicides are known to function by inhibiting the biosynthesis of methionine, which interferes with protein synthesis and prevents the secretion of the hydrolytic enzymes essential to the infection process of plant pathogenic fungi [7]. In addition, this class of fungicides is also known to inhibit cell division, and consequently can prevent both host penetration and the development of disease [8]. However, the extensive use of anilinopyrimidine fungicides has already resulted in the emergence of fungicide resistance in both *Botrytis cinerea* and *Penicillium expansum* [9,10,11], and it is regarded as at medium risk for fungicide resistance by the Fungicide Resistance Action Committee (FRAC).

Previous studies have shown that pyrimethanil is highly effective in inhibiting the conidial germination and germ tube elongation of *P. digitatum* [12] as well as the mycelial growth of *B. cinerea* [13], while our studies have found strong antifungal activity in *S. sclerotiorum*. One study has found that the cyprodinil class of anilinopyrimidines fungicide not only inhibited methionine biosynthesis but also suppressed cystine and cysteine biosynthesis [14]. However, the potential for pyrimethanil resistance in *S. sclerotiorum*, as well as possible resistance mechanisms, have yet to be fully investigated in detail. The objectives of the current study were therefore to (1) evaluate the fitness of pyrimethanil-resistant mutants; (2) investigate the molecular mechanism underlying pyrimethanil resistance in *S. sclerotiorum*; (3) assess the potential for cross-resistance between pyrimethanil and other commonly used fungicides; and (4) examine changes in the binding affinity of SsCGSs to pyraclostrobin through homology modeling and molecular docking.

## 2. Materials and Methods

### 2.1. Isolates and Fungicides

The four pyrimethanil-sensitive isolates used in the current study (DDJH-Pyri-S1, DDJH-Pyri-S2, DDJH-Pyri-S3, and DDJH-Pyri-S4, which had effective concentration for 50% inhibition (EC_50_) values of 0.411, 0.539, 0.610, and 0.513 μg/mL) were originally collected from soybean plants exhibiting typical symptoms of SSR infection growing in the fields of the Heilongjiang Province of China (Table 1). Four genetically stable pyrimethanil-resistant mutants (DDJH-Pyri-R1, DDJH-Pyri-R2, DDJH-Pyri-R3, and DDJH-Pyri-R4, which had EC_50_ values of 7.247, 14.526, 24.718, and 13.840 μg/mL) were generated by repeated exposure of the four parental isolates listed above to pyrimethanil under laboratory conditions (Table 1) following the method of previous studies [15]. All of the *S. sclerotiorum* mutants and parental isolates were routinely maintained on potato dextrose agar (PDA, 200 g/L potato, 20 g/L dextrose, and 20 g/L agar) medium at 25 °C.

The majority of the technical-grade fungicides used in the study, including 95.0% pyrimethanil, 97.0% prochloraz, 97.0% boscalid, 98.1% cyprodinil, and 97.5% pyraclostrobin (Kangbaotai Fine Chemical Co., Ltd., Wuhan, China), 96.0% fludioxonil (Hubei Jianyuan Chemical Co., Ltd., Wuhan, China), 96.2% tebuconazole (Sheyang Huanghai Pesticide Chemical Co., Ltd., Sheyang, China), and 95.0% fluazinam (Zhejiang Heben Pesticide & Chemicals Co., Ltd., Wenzhou, China), were dissolved in acetone. While the 98.1% carbendazim (Haili Guixi Chemical Co., Ltd., Yingtan, China) was dissolved in 0.1 mol/L hydrochloric acid. To keep the fungicide at its best, the resulting 10,000 μg/mL stock solutions were stored at 4 °C for no more than 2 weeks before being used to prepare serial dilutions for use in the various experiments. Due to solubility differences, two solvents had to be used for mycelial growth assays, in which neither solvent affected *S. sclerotiorum* at the concentration range used.

### 2.2. Biological Characteristics of Pyrimethanil-Resistant S. sclerotiorum Mutants

#### 2.2.1. Mycelial Growth

The mycelial growth of the four wild-type strains (DDJH-Pyri-S1, DDJH-Pyri-S2, DDJH-Pyri-S3, and DDJH-Pyri-S4), as well as the laboratory mutants (DDJH-Pyri-R1, DDJH-Pyri-R2, DDJH-Pyri-R3, and DDJH-Pyri-R4) was assessed using an assay described previously [15]. Briefly, mycelial plugs (5 mm in diameter) excised from the edge of 2-day-old colonies were transferred to fresh PDA medium and incubated at 25 °C with a 12 h photoperiod. The resulting colonies were measured at 24 h post inoculation (hpi). Six independent replicate plates were assessed for each isolate, and the entire experiment was performed twice.

#### 2.2.2. Sclerotia Production on PDA Medium

The sclerotia production of the four pyrimethanil-resistant resistant mutants and parental *S. sclerotiorum* isolates were assessed using the protocol of Wang [6], with a few minor modifications. In the current study, mycelial plugs (5 mm) were transferred to fresh PDA plates, and incubated at 25 °C for an extended period of 21 days, at which point, the number of sclerotia was recorded, and their fresh and dry weight (50 °C for 2 days) measured. Each isolate was represented by eight replicate plates, and the entire experiment was performed twice.

#### 2.2.3. Pathogenicity on Detached Tomato Leaves

The pathogenicity of the four pyrimethanil-resistant mutants were compared to that of their sensitive parental isolates using detached tomato leaves (Cultivar Zhongza 12, Host of *S. sclerotiorum*). Leaves of a similar size and equivalent growth stage were excised and rinsed with sterile water, before being air-dried in a laminar flow cabinet and transferred to Petri dishes (90 mm) lined with wet blotting paper to maintain high humidity. The leaves were inoculated with mycelial plugs (5 mm) cut from the margins of 3-day-old colonies, which were placed on the adaxial surface of the leaves. After inoculation, the leaves were transferred to a growth chamber and incubated at 25 °C. The resulting lesions were measured at 72 hpi in accordance with the method used in previous studies [6,15]. Each isolate was represented by at least eight individual leaves, and the entire experiment was performed twice (negative controls: without mycelial plugs; positive controls: sensitive parental isolates mycelial plugs).

### 2.3. Cloning and Sequence of SsCGS1 and SsCGS2 Genes

Fresh mycelium was collected from 200 mL PDB cultures of the various *S. sclerotiorum* mutant and parental isolates, and the genomic DNA was extracted following the method of previous studies [6,16]. Full-length sequences of the candidate genes (*SsCGS1*: *SS1G_11000*; *SsCGS2*: *SS1G_09924*) were then amplified using two primer sets designed according to previous study [17], which had the following sequences: SS1G_11000-F/SS1G_11000-R, ATGTCCGTCATCGAACTTGGAGAG/TCAAGACGTTGAATCTGCCGCAGC; SS1G_09924-F/SS1G_09924-R, TTCCAATCCCAACTTCATCC/ACAACGAGCCACCTCCACCT, corresponding to CGS1 and CGS2, respectively. The PCR reactions were performed using 50.0 μL reaction mixtures containing 25.0 μL 2.0 × ES Taq Master Mix, 1.5 μL template DNA, 2.0 μL each primer, and 21.5 μL ultrapure water, which were prepared in accordance with the instructions from the PCR kit (Jiangsu Cowin Biotech Co., Ltd., Taizhou, China), and processed using a 96-well thermal cycler (Applied Biosystems, Thermo Fisher Scientific, Waltham, MA, USA), with the following program: melting at 94 °C for 5 min; followed by 35 cycles of 94 °C for 30 s, 57 °C for 30 s, and 72 °C for 1.5 min; and a final extension at 72 °C for 10 min. The resulting PCR products were purified, and cloned into the pMD_19-T vector using a cloning kit (TaKaRa, Biomedical Technology (Beijing) Co., Ltd., Beijing, China) before being sequenced commercially (Wuhan Gene-create Biotechnology Co., Ltd., Wuhan, China). The resulting sequence data were analyzed using the DNAMAN software package (Ver. 8.0; Lynnon Biosoft, San Ramon, CA, USA), and multiple sequences alignments of the predicted amino acid sequences prepared to identify amino acid differences between the resistant and sensitive isolates as described previously [6,15].

### 2.4. Comparison of SsCGS1 and SsCGS2 Expression Levels in Pyrimethanil-Resistant Mutants of S. sclerotiorum

Total RNA was extracted from fresh mycelium samples using a fungal RNA kit (Omega Bio-Tek, Norcross, GA, USA) following the instructions of the manufacturer, and used as a template for quantitative real-time PCR (qPCR) with gene specific primers sets designed to amplify partial sequences of the *S. sclerotiorum SsCGS1* and *SsCGS2* genes, including RT-SS1G_11000-F/RT-SS1G_11000-R and RT-SS1G_09924-F/RT-SS1G_09924-R, which had the following sequences, GATCCCATCCCTTGGTAAAGTC/AAGTCCACCATAACCTCCATTC and TGATGCAAAGACGAGGAAGG/AGCTCGGCTCAAATCATCTATC, as well as DDJH-RT-actin-F/DDJH-RT-actin-R: ATGCGGTTGAGTTCTGCGTGT/CATGTCAACACGAGCAATG, which were designed to amplify the actin reference gene. First-strand cDNA was initially prepared using the PrimeScript RT kit (TaKaRa, Kusatsu, Japan), while the qPCR amplification was performed using the Quantstu Dio6 Flex PCR Detection System (Thermo Fisher, Waltham, MA, USA) in conjunction with SYBR Green I fluorescent dye. The relative expression of each gene was calculated using the method from a previous study [6] with β-tubulin being used as the reference gene [15]. The study evaluated three independent biological replicates for each mutant in order to calculate the mean and standard error.

### 2.5. Molecular Docking

To evaluate the binding affinity of pyrimethanil with four types of SsCGS1 (DDJH-Pyri-WT, DDJH-Pyri-R1, DDJH-Pyri-R3, and DDJH-Pyri-R4) and two types of SsCGS2 (DDJH-Pyri-WT and DDJH-Pyri-R1), molecular docking was conducted using Alphafold software (https://alphafoldserver.com/welcome, accessed on 23 January 2025). The three-dimensional structure of the pyrimethanil for docking is derived from the PubChem database (PubChem CID: 916503). Based on the Global Model Quality Estimate (GMQE) values, the optimal protein model was selected for molecular docking with pyrimethanil. AutoDockTools software (Version 1.5.7) was used to evaluate the binding affinity between the target protein and pyrimethanil. The smaller the binding energy, the stronger the affinity between the protein and the ligand. The results were visualized using PyMOL software (Version 2.5).

### 2.6. Cross-Resistance of Pyrimethanil with Other Fungicides

The potential for cross-resistance between pyrimethanil and eight commonly used fungicides, including carbendazim, fludioxonil, prochloraz, tebuconazole, pyraclostrobin, boscalid, fluazinam, and cyprodinil, was assessed using the mycelial growth assay, according to the previously described methods [16]. The EC_50_ values were determined by growing the pyrimethanil-resistant mutants as well as the sensitive parental isolates on PDA plates with a range of fungicide concentrations (factorial arrangement) as follows: 0.2, 0.4, 0.8, 1.6, 3.2, 6.4, 12.8, 25.6, 51.2, and 102.4 μg/mL for pyrimethanil with the resistant mutants; 0.05, 0.1, 0.2, 0.4, 0.8, 1.6, 3.2, and 6.4 μg/mL for carbendazim, as well as pyrimethanil with the sensitive isolates; and 0.0625, 0.0125, 0.025, 0.05, 0.1, 0.2, 0.4, and 0.8 μg/mL for cyprodinil, fludioxonil, prochloraz, fluazinam, tebuconazole, pyraclostrobin, and boscalid, respectively. Each isolate/fungicide/concentration combination was represented by at least three replicate plates, and the entire experiment was performed three times.

### 2.7. Data Analysis

The data collected in the current study were first subjected to ANOVA using SPSS software (Ver. 17.0; SPSS Inc., Chicago, IL, USA), before statistical differences between treatments were determined using Fisher’s least significant difference test (ɑ = 0.05). Linear regression analysis was carried out using SPSS software for the calculation of the EC_50_ value.

## 3. Results

### 3.1. Biological Characteristics of Four Pyrimethanil-Resistant Mutants of S. sclerotiorum

**Figure 1:**
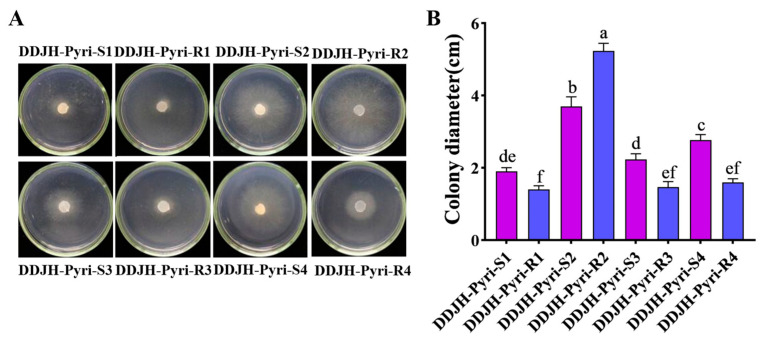
Mycelial growth of four pyrimethanil-resistant mutants of *S. sclerotiorum* compared to their sensitive parental isolates. The panels (**A**) show the colony growth of both the resistant mutants (R) and sensitive parental isolates (S) on PDA medium after 24 hour’s incubation at 25 °C, while the graph (**B**) shows the average colony diameter. Different letters above the columns indicate significant differences according to Fisher’s least significant difference test (*p* = 0.05).

**Figure 2:**
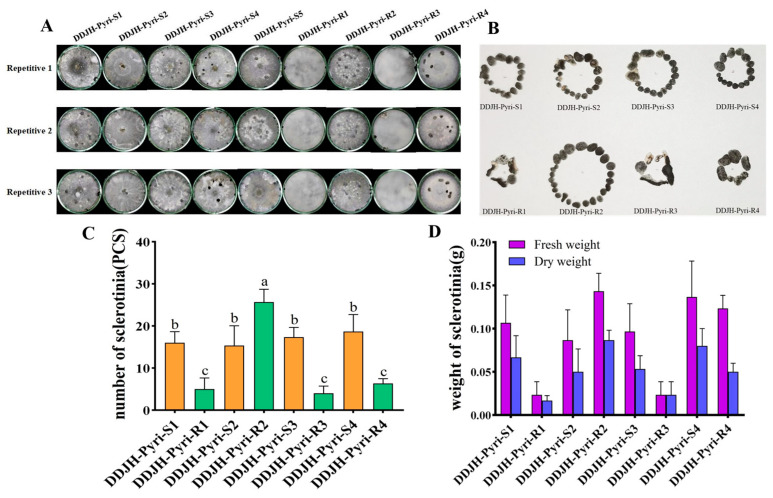
Sclerotia production of four pyrimethanil-resistant mutants of *S. sclerotiorum* compared to their sensitive parental isolates. The top panels show the colonies of both the resistant mutants (R) and sensitive parental isolates (S) after 21 days incubation on PDA medium at 25 °C (**A**), as well as the sclerotia from each plate (**B**), while the graphs below show the number (**C**) and weight (**D**), both fresh and dry, of the sclerotia collected. Different letters above the columns indicate significant differences according to Fisher’s least significant difference test (*p* = 0.05).

**Figure 3:**
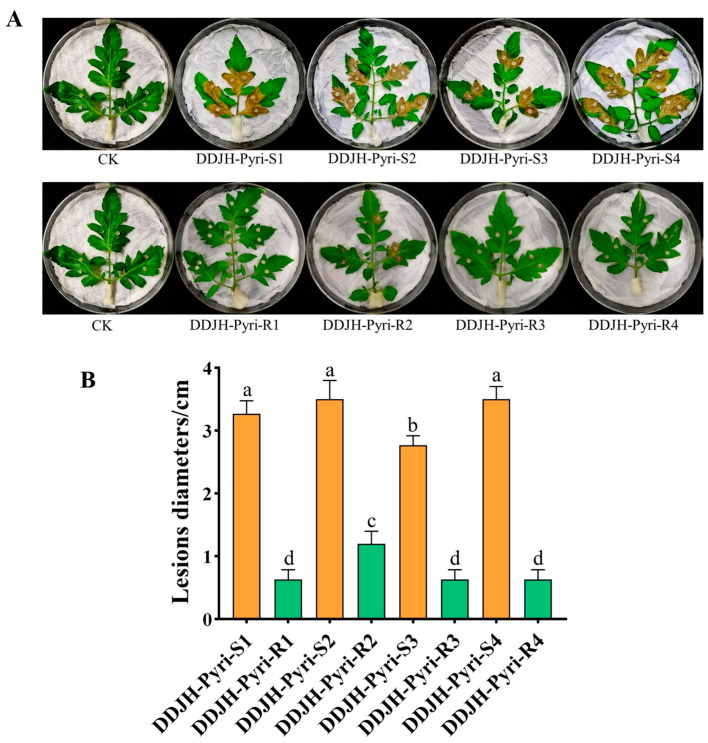
Pathogenicity of four pyrimethanil-resistant mutants of *S. sclerotiorum* compared to their sensitive parental isolates. The panels (**A**,**B**) show the lesions produced by both the resistant mutants (R) and sensitive parental isolates (S) on detached tomato leaves at 72 hpi. Different letters above the columns indicate significant differences according to Fisher’s least significant difference test (*p* = 0.05).

The mycelial growth of all the pyrimethanil-resistant mutants were significantly (*p* < 0.05) altered compared to their parental isolates, with DDJH-Pyri-R2 exhibiting increased growth, and three mutants (DDJH-Pyri-R1, DDJH-Pyri-R3, and DDJH-Pyri-R4) having reduced growth at 24 hpi (Figure 1). Similarly, the resistant mutants generally exhibited reduced sclerotia production, especially when the dry weight was considered, with the most affected mutants (DDJH-Pyri-R1 and DDJH-Pyri-R3) exhibiting as much as 90% reduction in sclerotia weight, although one mutant (DDJH-Pyri-R2) broke this trend with significantly increased sclerotia production (Figure 2). In addition, the pyrimethanil-resistant mutants were also found to have significantly (*p* < 0.05) reduced pathogenicity on detached tomato leaves (Figure 3), with one producing lesions approximately 40% smaller than its parental isolate, while three mutants (DDJH-Pyri-R1, DDJH-Pyri-R3, and DDJH-Pyri-R4) completely failed to produce an infection even at 72 hpi. Taken together, these results indicate that pyrimethanil resistance generally results in a cost to fitness, but that in some cases it can result in increased growth and sclerotia production. Such variation amongst the different mutants might also suggest that different mechanisms of resistance might be responsible.

### 3.2. Sequence Analysis of the SsCGS1 and SsCGS2 Protein in Pyrimethanil-Resistant Mutants of S. sclerotiorum

The DNA sequences of the *SsCGS1* and *SsCGS2* genes (GenBank: *SS1G_11000* and *SS1G_09924*) of the pyrimethanil-resistant mutants were compared to those of the sensitive isolates, which identified several point mutations that might be associated with pyrimethanil resistance (Table 2). For example, the pyrimethanil-resistant mutant DDJH-Pyri-R1 contained four mutations in the predicted sequences of its cystathione-γ synthase (CGS), which is encoded by the SsCGS1 protein, including the substitution of glycine for the valine at residue 117 (V117G), histidine for tyrosine at residue 285 (Y285H), histidine for asparagine at residue 421 (N421H), and alanine for valine at residue 490 (V490A), as well as one change in its cystathione-β lyase (CBL), which is encoded by SsCGS2, that resulted in the substitution of arginine for glycine at residue 289 (G289R). Three of the other pyrimethanil-resistant mutants were also found to have amino acid changes in their predicted CGS sequences, although in each case only a single residue was affected: M457T in DDJH-Pyri-R3 and H487R in DDJH-Pyri-R4. In contrast to these three mutants, the pyrimethanil-resistant mutant, DDJH-Pyri-R2, was conspicuous in having no mutations at all in either of its SsCGS1 protein, which suggests that an alternative resistance mechanism might be responsible for the observed resistance of this mutant. This observation, along with the fact that DDJH-Pyri-R2 lacked mutations in its SsCGS protein, might also explain why the four mutants exhibit such distinct biological characteristics.

### 3.3. Relative Expression of the SsCGS1 and SsCGS2 Genes in Pyrimethanil-Resistant Mutants of S. sclerotiorum

**Figure 4:**
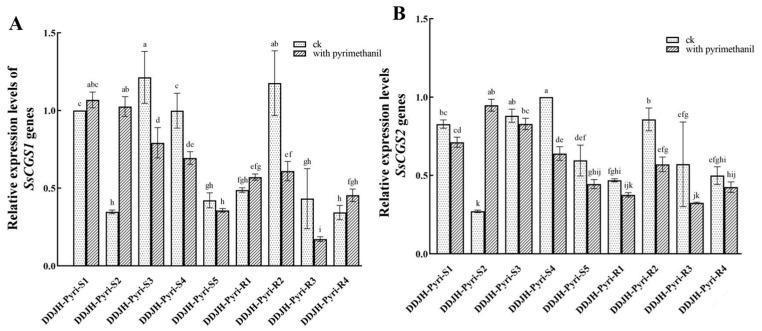
Relative expression of the *SsCGS1* and *SsCGS2* genes in four pyrimethanil-resistant mutants of *S. sclerotiorum* compared to their sensitive parental isolates. The relative expression of the *SsCGS1* gene (**A**), which encodes a cystathione-γ synthase (CGS) and *SsCGS2* (**B**), which encodes cystathione-β lyase (CBL), was assessed in resistant mutants (R) and sensitive parental isolates (S) in both the absence and presence of pyrimethanil (0.1 μg/mL), using actin as the reference gene. Different letters above the columns indicate significant differences according to Fisher’s least significant difference test (*p* = 0.05).

The qPCR analysis conducted in the current study found that the presence of pyrimethanil resulted in the altered expression of the *SsCGS1* and *SsCGS2* genes in all of the mutants and parental isolates investigated (Figure 4). In generally, the fungicide caused a significant (*p* < 0.05) reduction in the expression of both genes in the wild-type parental isolates, although the fold change in expression was usually not that great, especially in the case of the *SsCGS2* gene. The one exception to this was the parental isolate DDJH-Pyri-S2, which exhibited dramatically increased expression in the presence of pyrimethanil. In addition, it is interesting to note that this isolate had significantly lower basal expression of both genes in the absence of the fungicide, when compared to the other three parental isolates. A similar pattern of expression was observed in the pyrimethanil-resistant mutants. In this case, all of the mutants exhibited reduced expression of their *SsCGS2* gene in the presence of the fungicide. Again, the fold change in expression caused by the presence of pyrimethanil was generally rather small, with some mutants (DDJH-Pyri-R1 and DDJH-Pyri-R4) actually exhibiting a slight increase in the expression of their *SsCGS1* gene. It was also noted that in general the mutants exhibited lower expression of both genes (an approximate 50% reduction) compared to their parental isolates. However, in contrast, one mutant, DDJH-Pyri-R2, exhibited a quite different pattern of expression compared to the others, and indeed to its parental isolate. In this case, the mutant had a dramatically higher basal level of expression (approximately 3-fold higher) of both genes compared to its parental isolate. Furthermore, unlike its parental isolate, DDJH-Pyri-S2, which exhibited dramatically increased expression in the presence of pyrimethanil, the resistant mutant DDJH-Pyri-R2, exhibited significantly (*p* < 0.05) reduced expression of both *SsCGS* genes. This result is consistent with the hypothesis that DDJH-Pyri-R2, which had no amino acid changes in its *SsCGS* sequences, employs a different mechanism of resistance than the other mutants, and suggests that in this case, resistance might result from changes to the regulation of the *SsCGS* and CBL proteins, rather than changes to their structure. Meanwhile, the altered expression of the parental isolates in response to pyrimethanil, and the suppressed expression of the mutants that did have amino acid changes in their *SsCGS* sequences, indicates that the *SsCGS* genes play an important role in the response of parental *S. sclerotiorum* isolates to treatment with pyrimethanil, and that the depressed levels of *SsCGS1* and *SsCGS2* expression observed in the mutants might also contribute to their reduced sensitivity to the fungicide treatment.

### 3.4. Molecular Docking Analysis

**Figure 5:**
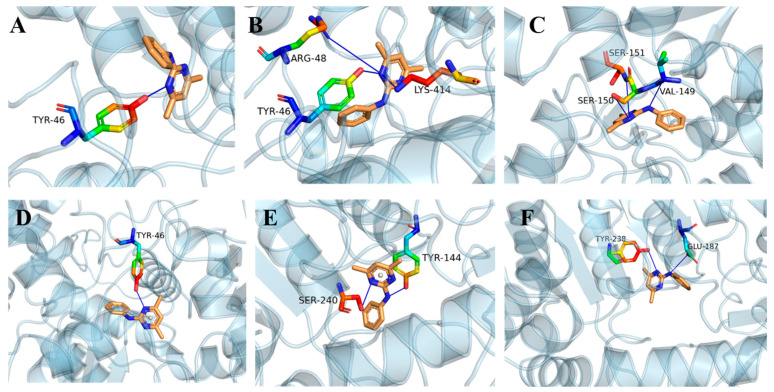
Molecular docking analysis of pyrimethanil with six types of SsCGS. (**A**–**D**) binding model of SsCGS1-WT, SsCGS1-R1, SsCGS1-R3, SsCGS1-R4 and pyrimethanil, respectively; (**E**,**F**) binding model of SsCGS2-WT, SsCGS2-R1 and pyrimethanil.

The three-dimensional protein models of four types of SsCGS1 and two types of SsCGS2 in *S. sclerotiorum* were constructed using AlphaFold (Version 2) based on the CGSs structure of *Phialocephala subalpina* (AlphaFold Datebase model of A0A1L7WXE3_9HELO) and *Botryotinia fuckeliana* (AlphaFold Datebase model of G2Y0L9_BOTF4). The GMQE values of SsCGS1 of four models and SsCGS2 of two models were 0.87 and 0.88, respectively. The sequence identities to SsCGS1-WT, SsCGS1-R1, SsCGS1-R3, SsCGS1-R4, SsCGS2-WT, and SsCGS2-R1 were 77.32, 76.82, 77.32, 77.15, 96.73, and 96.51%, respectively. The docking results indicated that binding patterns of pyrimethanil with six types of SsCGSs in *S. sclerotiorum* were different (Figure 5), and the mutation of SsCGSs altered the binding site of pyrimethanil. The calculated binding energies were −6.4 kcal/mol for SsCGS1-WT and pyrimethanil, and SsCGS1-R3 interaction and three hydrogen bonds formed at VAL149, SER150, and SER151; −4.7 kcal/mol for pyrimethanil and SsCGS1-R4 interaction and one hydrogen bond formed at TYR46; −6.3 kcal/mol for pyrimethanil; while SsCGS1-R1 interaction and three hydrogen bonds formed at TYR46, ARG48 and LYS414, −6.7 kcal/mol for pyrimethanil. Furthermore, a binding energy of −6.5 kcal/mol for SsCGS2-WT protein and pyrimethanil, while a binding energy of −1.8 kcal/mol was observed for SsCGS2-R1 and pyrimethanil, and two hydrogen bonds formed at GLU187 and TYR238. These findings suggest that the binding site mutations in SsCGSs result in a reduced affinity toward pyrimethanil. However, SsCGS1-R1 mutation causes increased affinity compared to the SsCGS1-R3, SsCGS1-R4, and SsCGS2-R1.

### 3.5. Cross-Resistance Between Pyrimethanil and Other Fungicides

The current study found no evidence of cross-resistance between pyrimethanil and any of the other fungicides tested, which included carbendazim, fludioxonil, prochloraz, tebuconazole, pyraclostrobin, boscalid, fluazinam, and cyprodinil (Table 1). This result indicates that in addition to providing effective protection from *S. sclerotiorum*, pyrimethanil could be useful in mitigating the risk of resistance emerging to other fungicides that have different modes of action.

## 4. Discussion

Pyrimethanil is an aniline-based pyrimidine fungicide that interferes with the biosynthesis of methionine and inhibits the activity of extracellular hydrolase enzymes in plant pathogens. Although pyrimethanil is widely used in the prevention and control of gray mold [7,18], FRAC has classified similar anilinopyrimidine fungicides as at moderate risk of fungicide resistance [19], and as such, measures to ensure their correct application are required to prevent the occurrence of resistance.

In fact, repeated and inappropriate use has already resulted in numerous reports of resistance emerging in field populations of plant pathogens. For example, pyrimethanil-resistant strains of *Alternaria alternata* were collected from the pistachio orchards of California between 1998 and 2003 [20], while resistant isolates of *P. expansum* were reported in the apple warehouses of Pennsylvania in 2011 [21]. In China, resistant isolates of *Botrytis cinerea* were detected in tomato crops growing in Henan Province between 2013 and 2014 [22], while Zhang et al. reported that the frequency of pyrimethanil resistance in *Botrytis cinerea* populations in the vineyards of China ranged from 22.22% to 62.50%, and more worryingly that most isolates exhibited a moderate or highly resistant phenotype, with up to 44.23% being highly resistant [23]. This might be related to the excessive frequency of using fungicides or their unscientific and unreasonable application. Although pyrimethanil has been used as an agricultural fungicide in China for many years, it has yet to be registered for the control of SSR (http://www.chinapesticide.org.cn/zwb/dataCenter, accessed on 1 January 2025), and given previous experience, further investigation is required to assess the risk of expanding its use. The results of the current study confirmed that pyrimethanil exhibited strong antifungal activity against *S. sclerotiorum*, the causal organism of SSR (Table 1). Meanwhile, examination of four highly pyrimethanil-resistant laboratory mutants indicated that resistance usually came at some cost to fitness with regard to growth rate, sclerotia production, and pathogenicity, which was similar to the results of a previous study that found resistance to the anilinopyrimidine fungicide, cyprodinil, also resulted in reduced sclerotia production and pathogenicity in *S. sclerotiorum* [14].

As reports of resistance to aniline-based pyrimidine fungicides have increased over the years, research has begun to focus on characterizing potential resistance mechanisms. At present, it is generally believed that the mechanism of resistance in plant pathogenic fungi centers around amino acid mutation in the primary structure of the cystathione-γ synthase (CGS) and cystathione-β lyase (CBL) enzymes play a key role in the methionine synthesis pathway [18]. For example, a previous study concluded that substitutions at residues 30, 488, and 588 in the CGS of laboratory mutants of *Aspergillus flavus* were associated with the development of pyrimethanil resistance in this species [24]. Meanwhile, amino acid changes at residues in the CBL sequences of *S. sclerotiorum* and *B. cinerea* were associated with resistance to the fungicides cyprodinil and pyrimethanil, respectively [13,14]. However, there have also been reports of alternative resistant mechanisms, with one study indicating that after exposure to fluopyram, amino acid in FgSDHC substitution A73V made the gene significantly upregulated [25]. These previous observations indicate that there is no single resistance mechanism that confers pyrimethanil resistance to plant pathogens, but rather that a range of different mechanisms can be responsible. The current study found further evidence of this hypothesis. In this case, four of the resistant mutants of *S. sclerotiorum* were found to have point mutations in their *SsCGS1* gene that resulted in changes to the amino acid sequence of their *SsCGS* proteins. However, the study failed to identify a single conserved mutation responsible for pyrimethanil resistance, with each mutant having amino acid changes at different residues (Table 2). In addition, it was also found that one of the pyrimethanil-resistant mutants had a second point mutation in its *SsCGS2* gene, which resulted in an amino acid change to its CBL sequence. These results therefore provide additional evidence that amino acid changes in fungal SsCGS, and SsCBL sequences can lead to resistance to pyrimethanil, although further studies, including site-directed mutagenesis of these proteins in parental isolates, are required to confirm these preliminary results. It was also interesting to note that the current study found evidence of an alternative resistance mechanism in *S. sclerotiorum*, as one mutant (DDJH-Pyri-R2) exhibited no changes to either its SsCGS, or SsCBL sequence, but instead, a dramatic change to the expression levels of the encoding genes (*SsCSG1* and *SsCSG2*) in comparison to the sensitive parental isolate (Figure 4), which might indicate that resistance resulted from a change in the regulation of the two genes, rather than changes to the protein structure. Although this hypothesis requires further verification, the results of the expression analysis also found that exposure to pyrimethanil generally caused a reduction in the expression of the two *SsCGS* genes in the parental isolates, and that the other mutants generally exhibited reduced expression compared to their parental isolates. Although such down-regulation has previously been observed for the *SsCSG1* gene in cyprodinil-resistant mutants of *S. sclerotiorum* [14], further research is required to determine whether these changes in expression of the *SsCGS* genes do indeed contribute to tolerance of pyrimethanil, or whether they are, rather, a consequence of other cellular changes associated with the primary resistance mechanism.

Although pyrimethanil has been widely used for the prevention and control of gray mold, there have been few reports regarding the potential for cross-resistance between pyrimethanil and other fungicides commonly used in agricultural production [23]. It was, therefore, encouraging that the current study found no evidence of cross-resistance between pyrimethanil and the other fungicides assessed, including the anilinopyrimidine fungicide, cyprodinil, which indicates that in addition to providing effective control of *S. sclerotiorum*, pyrimethanil could be a useful compound for mitigating the risk of resistance emerging to these other fungicides that have different modes of action. As such, the current evidence suggests that the use of pyrimethanil in combination or alternation with carbendazim, fludioxonil, prochloraz, tebuconazole, pyraclostrobin, boscalid, fluazinam, and cyprodinil could provide effective control of SSR, while minimizing the risk of fungicide resistance. However, given the complex nature of pyrimethanil resistance, which appears to result from several different mechanisms, it is also necessary to carry out routine monitoring for the emergence of pyrimethanil-resistant populations of *S. sclerotiorum* in order to ensure that these fungicides are used appropriately to provide sustainable control of SSR in soybean crops.

## 5. Conclusions

Pyrimethanil-resistant *S. sclerotiorum* mutants exhibited significantly reduced mycelial growth, sclerotia production, and pathogenicity compared to their wild-type parental isolates, indicating that pyrimethanil resistance suffered a fitness penalty in *S. sclerotiorum*. Notably, molecular docking and point mutations experiments indicated amino acid changes in the predicted sequence of cystathione-γ synthase (CGS) and cystathione-β lyase (CBL), encoded by *SsCGS1* and *SsCGS2,* suggesting an alternative resistance mechanism. In addition, no cross-resistance was observed between pyrimethanil and other commonly used fungicides, including carbendazim, fludioxonil, prochloraz, tebuconazole, pyraclostrobin, boscalid, fluazinam, and cyprodinil, suggesting that pyrimethanil has significant potential for controlling soybean sclerotinia stem rot (SSR) caused by *S. sclerotiorum*.

## Figures and Tables

**Table 1:** Cross-resistance between pyrimethanil and eight commonly used fungicides in four pyrimethanil-resistant mutants of *S. sclerotiorum*.

Fungicide	Sensitive Parental Isolates				Pyrimethanil-Resistant Mutants			
	DDJH-Pyri-S1	DDJH-Pyri-S2	DDJH-Pyri-S3	DDJH-Pyri-S4	DDJH-Pyri-R1	DDJH-Pyri-R2	DDJH-Pyri-R3	DDJH-Pyri-R4
Pyrimethanil	0.441 ^Z^	0.539	0.610	0.513	7.247	14.526	24.718	13.84
Cyprodinil	0.009	0.045	0.022	0.002	0.091	0.014	0.058	0.026
Fludioxonil	0.001	0.002	0.003	0.002	0.001	0.004	0.001	0.006
Prochloraz	0.001	0.001	0.001	0.002	0.004	0.003	0.002	0.011
Fluazinam	0.002	0.001	0.001	0.002	0.001	0.001	0.001	0.002
Tebuconazole	0.074	0.091	0.089	0.090	0.066	0.095	0.001	0.234
Pyraclostrobin	0.017	0.052	0.074	0.051	0.225	0.022	0.089	0.132
Carbendazim	0.131	0.399	0.117	0.069	0.061	0.679	0.293	0.534
Boscalid	0.185	0.167	0.214	0.169	0.032	0.002	0.007	0.039

^Z^ Values indicate the mean effective concentration (μg/mL) for 50% inhibition (EC_50_), and all the data in this table are from this study.

**Table 2:** Point mutations associated with *SsCGS1* and *SsCGS2* genes of *S. sclerotiorum* and other plant pathogens.

Genes	Mutants	Amino Acid Changes	Species/Fungicides/References
*SsCGS1*	DDJH-Pyri-R1	V117G, Y285H, N421H, V490A	*S. sclerotiorum*/Pyrimethanil/Current study
	DDJH-Pyri-R2	/ ^N^	*S. sclerotiorum*/Pyrimethanil/Current study
	DDJH-Pyri-R3	M457T	*S. sclerotiorum*/Pyrimethanil/Current study
	DDJH-Pyri-R4	H487R	*S. sclerotiorum*/Pyrimethanil/Current study
*SsCGS2*	DDJH-Pyri-R1	G289R	*S. sclerotiorum*/Pyrimethanil/Current study
	DDJH-Pyri-R2	/	*S. sclerotiorum*/Pyrimethanil/Current study
	DDJH-Pyri-R3	/	*S. sclerotiorum*/Pyrimethanil/Current study
	DDJH-Pyri-R4	/	*S. sclerotiorum*/Pyrimethanil/Current study
*CGS1/CGS2*	HBWH-10	/	*Penicillium digitatum*/Pyrimethanil/12
	JXGZ-92	/	*Penicillium digitatum*/Pyrimethanil/12
	JXGZ-59	/	*Penicillium digitatum*/Pyrimethanil/12
	JXGZ-177	/	*Penicillium digitatum*/Pyrimethanil/12
*CGS1/CGS2*	PR1	/	*Aspergillus flavus*/Pyrimethanil/24
	PR2	/	*Aspergillus flavus*/Pyrimethanil/24
	PR3	/	*Aspergillus flavus*/Pyrimethanil/24
	PR4	/	*Aspergillus flavus*/Pyrimethanil/24
	PR5	/	*Aspergillus flavus*/Pyrimethanil/24

^N^/ Indicates no data available.

## Data Availability

The original contributions presented in this study are included in the article. Further inquiries can be directed to the corresponding authors.
